# Angular Dependence of Solar Cell Parameters in Crystalline Silicon Solar Cells Textured with Periodic Array of Microholes

**DOI:** 10.1002/gch2.201900105

**Published:** 2020-06-04

**Authors:** Serra Altinoluk, Naveen Kumar, Emine Hande Ciftpinar, O. Demircioglu, Rasit Turan, Dragica Vasileska

**Affiliations:** ^1^ Department of Electrical and Electronics Engineering Mugla Sitki Kocman University Kotekli Mugla 48000 Turkey; ^2^ Center for Solar Energy Research and Applications (GUNAM) Middle East Technical University Cankaya Ankara 06800 Turkey; ^3^ School of Electrical Computer and Energy Engineering Arizona State University Tempe AZ 85287‐5706 USA; ^4^ Department of Physics Middle East Technical University Cankaya Ankara 06800 Turkey

**Keywords:** crystalline silicon, periodic hole texturing, reactive ion etching, solar cells

## Abstract

Surface texturing is an indispensable way of increasing absorption in solar cells. In order to properly characterize the effect of texturing, the angular dependence of the incidence light should be addressed. This is particularly important when the actual application where the incidence angle of the sunlight varies during the day is considered. This study presents the angular dependence of solar cell parameters in the case of periodically textured crystalline silicon (c‐Si) solar cells with microholes. A standard solar cell with pyramid texturing is also studied for comparison. It is shown that the incidence angle for the highest efficiency depends on the surface structure. While a standard pyramid‐textured surface performs best at the zero angle of incidence, it is needed to tilt the sample with microholes textures 15° with respect to the surface normal. This is also confirmed by the simulation study performed for the structures presented in this study.

## Graphical abstract

The efficiency of a solar cell strongly depends on the properties of the interaction between the incoming light beam and the surface of the device. This study shows the angular dependence of performance in a solar cell with various surface texture patterns. It is concluded that for each surface pattern there exists a different tilt, resulting in maximum cell performance.

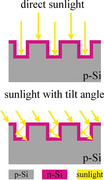

## 1 Introduction

**Figure 1:**
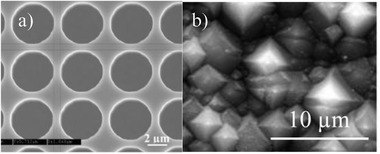
Scanning electron microscopy (SEM) images of textured surfaces: a) periodic hole texturing obtained with lithography and dry etching, and b) random pyramid texturing with wet etching, lithography‐free.

The characterization of c‐Si is carried out under standard test conditions (STC) specifying an operation temperature of 25 °C, irradiance of 1000 W m^−2^ with the air mass 1.5 (AM 1.5) spectrum. For STC sunlight beam hits directly on top of the surface without any tilt angle relative to the surface.^[^ ^1^, ^2^ ^]^ However, these conditions do not describe realistic situation; they only represent a small portion of actual conditions in which solar cells and panels operate in the application fields. In order to assess the performance of the solar cells under actual conditions, characterization techniques should be adopted depending on the medium of the device, the light intensity and the light incidence angle, physical location of the cell according to the duration of a day and climate.^[^ ^3^, ^4^, ^5^, ^6^ ^]^ Standard photovoltaic modules are ground fixed, but the sun’s position is changing in time depending on earth’s movement, so an angle resolved performance analysis is the only way to obtain actual field performance of the system.^[^ ^7^, ^8^ ^]^ This is particularly important when the surface of the cell is textured with a 3D structure such as pyramids, micro and nanorods, pillars, holes etc. Recently, a variety of such surface structures have been proposed and tested for a better light management on the surface of the solar cell.^[^ ^9^, ^10^, ^11^, ^12^, ^13^ ^]^ We have recently demonstrated fabrication of solar cell on Si wafer textured with periodic holes with micrometer (µm) size.^[^ ^14^ ^]^ In this case of a periodic hole structure shown in **Figure** **1** was fabricated by reactive ion etching (RIE) using photolithography. This structure resulted in better short circuit current performance as expected.^[^ ^15^, ^16^ ^]^ However, for a better understanding the device performance under actual condition, angular dependence of the measurement needs to be addressed.

**Figure 2:**
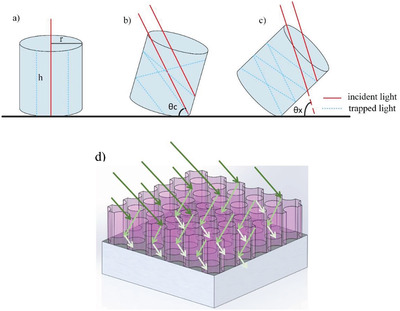
Schematic representation of hole textured surface under a) direct sunlight, no tilt angle, b) (θ_c_) critical angle, c) (θ_x_ > θ_c_) bigger than the critical angle. d) 3D representation of the trapped light’s bouncing inside the holes.

When light enters the holes on the surface, multiple reflection from the walls of the holes leads to higher absorption and thus an increase in the short circuit current density (*J*_sc_) values. This phenomenon is schematically illustrated in **Figure** **2**. In addition to the diameter and the depth of the holes, the angle incidence is also an important parameter in the penetration depth of the light. It is seen that the number of interactions with the wall initial increases with the incident angle. However, after a critical angle, the decrease in the total light flux crossing the surface becomes more dominant leading to a decrease in the overall performance of the cell.^[^ ^4^, ^5^ ^]^

In this paper, we report on the angular dependence of photovoltaic performance for devices having microholes as a texturing structure. We present both simulation results performed by SILVACO‐ATLAS program and experimental results. We show that for an accurate analysis of textured surface, the angular dependence should be taken into the account.

### 1.1 Experimental Procedures

**Figure 3:**
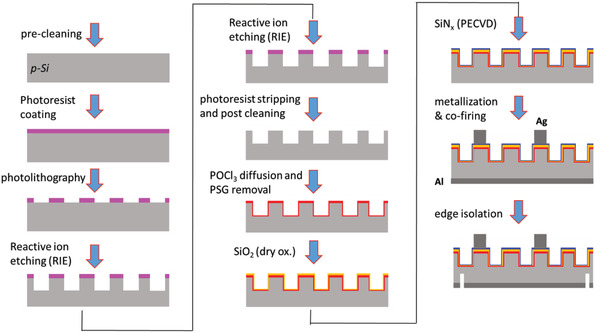
Process flow of solar cell fabrication with periodically textured front surface.

In this study, p‐type silicon wafers with <100> orientation having thickness of 525 µm and resistivity of 1–5 Ω cm were used as substrates. Microholes with varying dimensions and solar cell devices on these holes were fabricated by following the experimental procedures shown in **Figure** **3**. Photolithography and RIE process were optimized to obtain well defined microhole structure on the surface. The process steps were applied for the fabrication of standard Al‐BSF solar cells as shown in Figure 3.

Solar cells were then measured by an angle resolved measuring system where the incident angle was varied by tilting the sample in front of the collimated light of the solar simulator. In order to understand the measured angular dependence, we have also performed a simulation study using SILVACO‐ATLAS platform.

## 2 Silvaco Simulations

To simulate the solar cells with hole texturing, the commercial TCAD software Silvaco was used.^[^ ^17^ ^]^ Silvaco is a Device (ATLAS) and a Process (ATHENA) TCAD simulation tool, consisting of various modules to model semiconductor devices and their associated physical phenomena. Luminous (part of ATLAS) gives the absorption profiles for the device which are then converted into photogeneration profiles. The generation profiles are then used in the carrier continuity equations to calculate the IV‐characteristics of the solar cell. ATLAS provides four physical models for light propagation: Ray tracing (RT), transfer matrix method (TMM), beam propagation method (BPM), and finite difference time domain (FDTD) method. In our case, since the dimensions of the device are large compared to the wavelength of light, ray tracing model is the appropriate choice. RT ignores the interference and coherence effects in the propagation model. RT is computationally inexpensive, which makes it ideal for simulating thick solar cells.

Model Description: To properly model carrier transport in solar cells (carrier separation), it is necessary to incorporate Shockley Read Hall (SRH) and Auger recombination mechanisms in addition to optical generation which is already provided by Luminous. Mobility has been accounted for by using concentration dependent mobility and field dependent mobility models.

**Figure 4:**
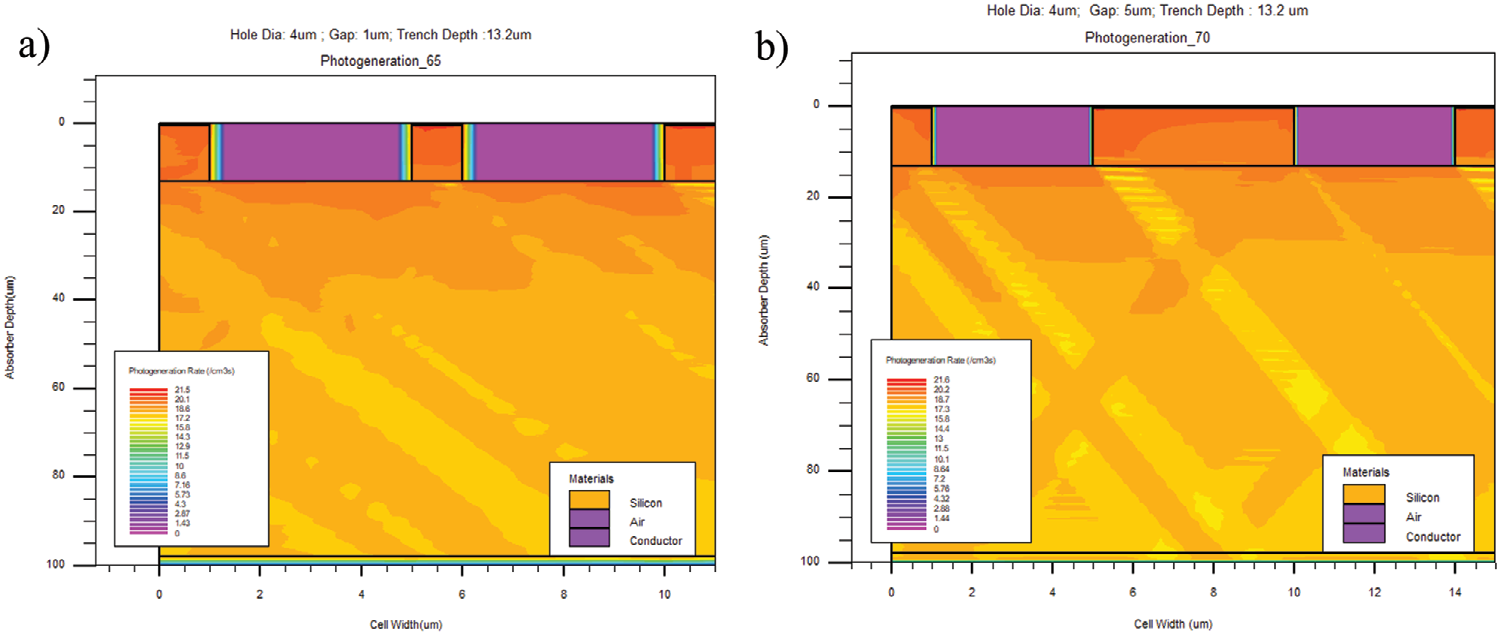
Photogeneration rate of the surfaces with 4 µm diameter and 13.2 µm depth: a) gap = 1 µm with the source at an angle of 25° from the horizontal, b) gap = 5 µm with the source at an angle of 20° from the horizontal.

Device Design: The array of trenches in the textured solar cell is approximated as rectangular holes in the 2D device design. **Figure** **4** shows the solar cell designed in Silvaco ATLAS.

The design shows surfaces textured with holes having 4 µm diameter and 1 and 5 µm gap values between the holes. The hole depth is 13.2 µm. The absorber thickness is limited to 100 µm instead of 500 µm to limit the number of mesh points (triangles) to be simulated, thus reducing the overall simulation time significantly. The simulation is then carried out by placing the beam for vertical incidence and changing the angle in steps of 5° up to 45° of incidence.

**Figure 5:**
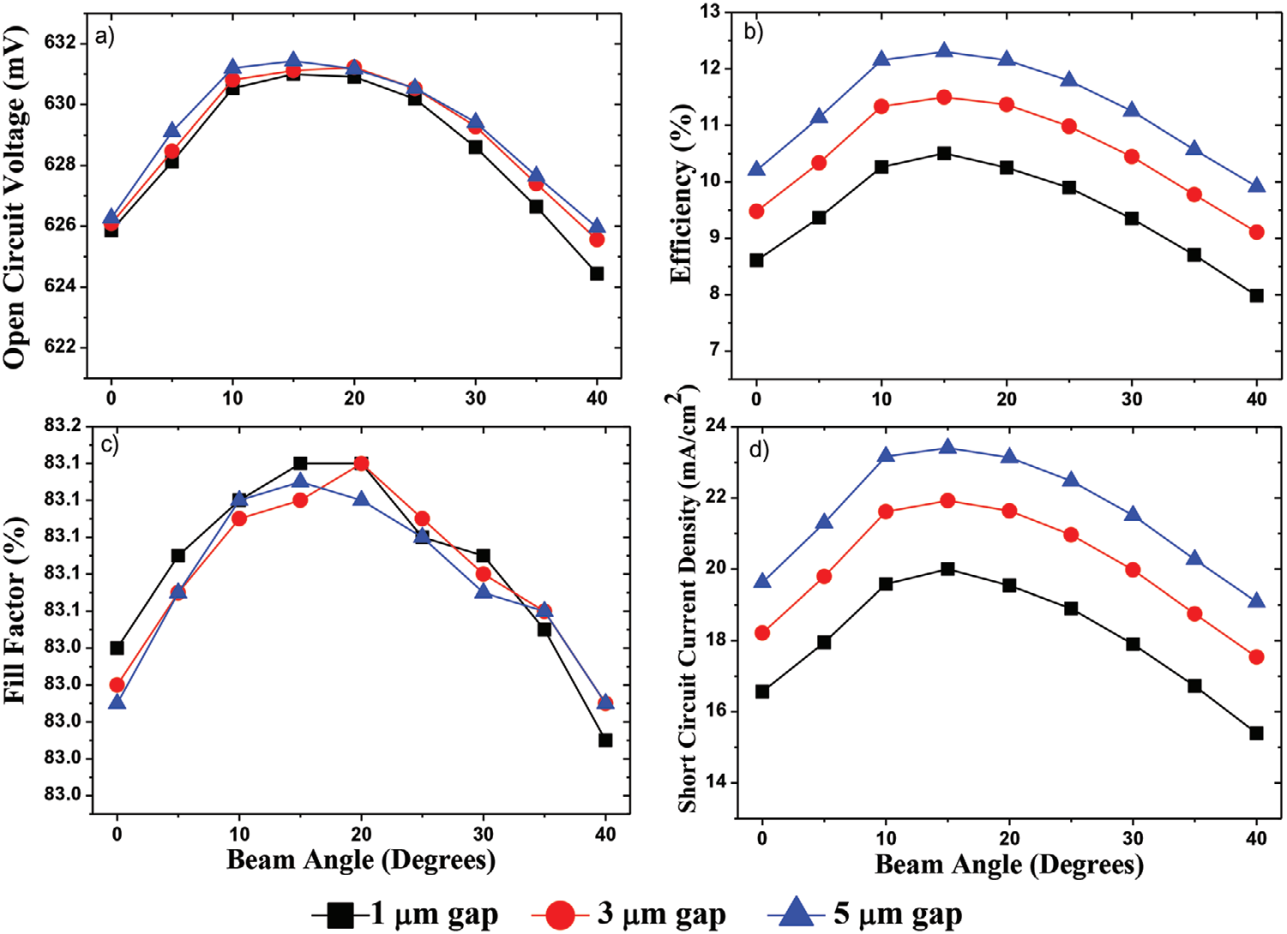
Simulation results obtained from the surfaces with 4 µm diameter 1, 3, 5 µm gap: a) open circuit voltage, b) efficiency, c) fill factor, d) short circuit current density.

The performance parameters of the cell were extracted from the simulation for two different pitch sizes, keeping the diameter and the hole depth constant. The plots of the various parameters versus the beam incident angle are shown in **Figure** **5**. As is evident from the plots, the performance of the cell tends to increase as the source is more and more inclined. *J*_sc_ reaches its maximum level at a beam incident angle of 15°and then falls off.

Limitations: The primary limitation in the simulations performed is the simulation of a cell with lesser absorber thickness (100 µm) instead of the dimension of the actual experimental device (500 µm). This results in a decreased value of the short circuit current and, hence, the efficiency of the simulated solar cell as compared to the experimental values. This limitation arises from the fact that as the beam angle increases, the number of reflections also increases. Thus, it becomes computationally prohibitive task in the case of large thicknesses, as the number of data points becomes huge. Although the simulated cell is thinner than the experimental cell, the trends are the same.

### 2.1 Angular Dependent Solar Cell Performances: Experimental Results

**Figure 6:**
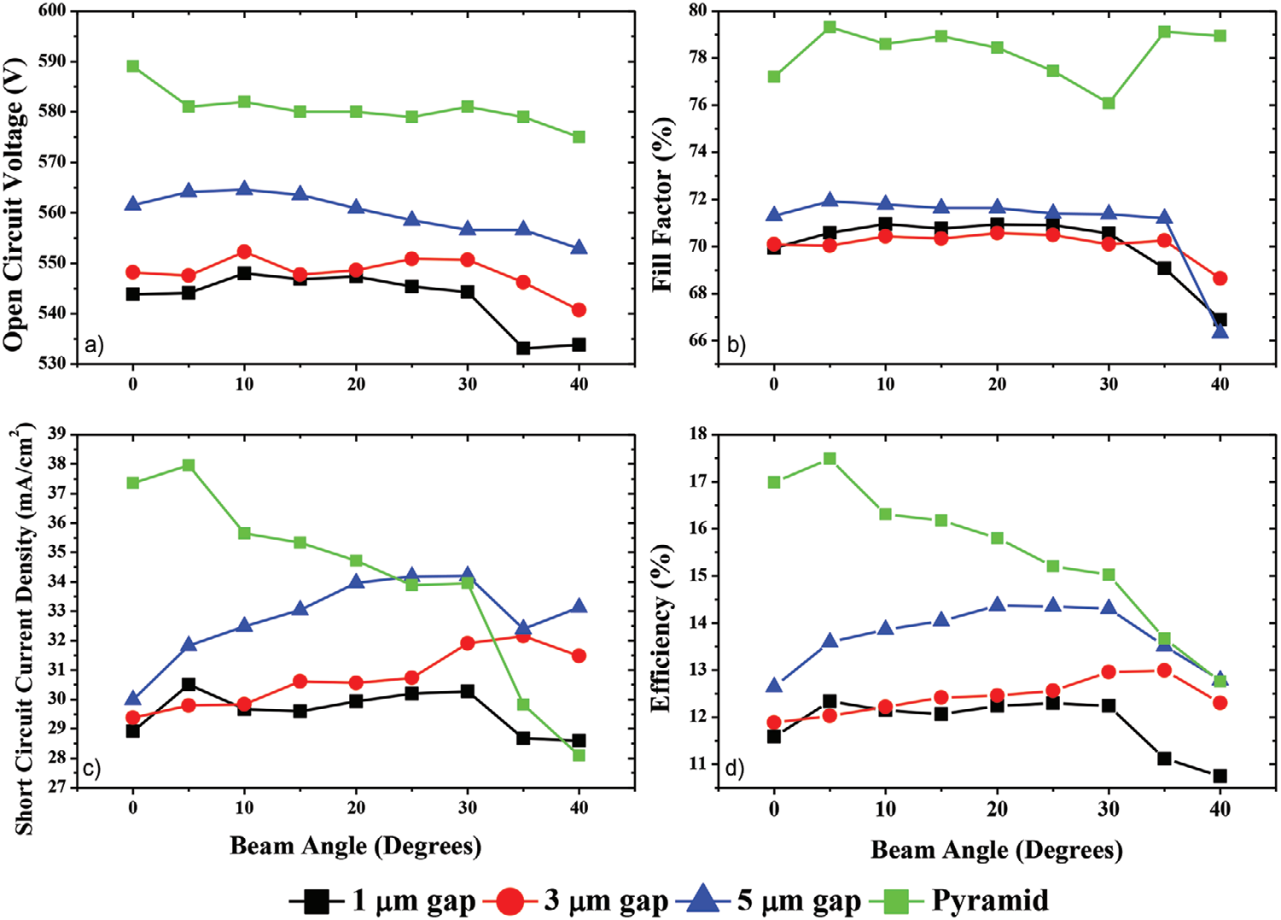
Solar cell performances between 0° to 45°: a) open circuit voltage, b) fill factor, c) short circuit current density, d) efficiency, obtained from the surfaces with 4 µm diameter 1, 3, 5 µm gap, fabricated with dry plasma etching, and random pyramid texturing obtained with wet chemical etching.

Solar cells that were fabricated on Si surface with different distribution of periodic holes were analyzed at incidence angles varying in between 0° and 45°. A reference cell with standard pyramid texturing was also measured for comparison. As seen in **Figure** **6** the pyramid texturing exhibits the highest efficiency when the incident angle is zero. The efficiency decreases with the angle almost monotonically as also reported by others previously.^[^ ^7^ ^]^ For the samples with microholes, the efficiency first increases with the incidence angle and then decreases as also predicted by the simulation results. Although it is hard to obtain a perfect match between SILVACO simulation and experimental data we observe a convincing and qualitative agreement between them.

It is seen that the microhole samples behave quite differently than pyramid textured samples and this feature have to be taken into the account for designing solar cells with hole textured surface. While a zero‐angle incidence is preferred in the case of standard pyramid textured solar cells, an incidence angle of around 15° is recommended for the microhole textured surfaces. The maximum efficiency value obtained from microhole samples displayed in Figure 6 is 14.3%, which is lower than the reference samples. The main reasons for this low performance are the increase in the surface area due to the microhole formation and contact resistance between metal layer and the textured surface. High recombination losses at the surface should be addressed and eliminated by a proper passivation technique which needs to be studied in a separate study. Similarly contact formation between Ag layer and the substrate should be studied carefully and optimized with respect to contact resistance of the junction. When properly optimized, the device performance as well as the agreement with the simulation result will improve.

## 3 Conclusions

Angular dependence of the performance of a solar cell textured with microholes has been studied theoretically and experimentally and compared with the standard pyramid textured solar cells. Pyramid textured solar cells have exhibited highest performance at the normal incidence as expected while samples with microholes as the texturing features on the surface has shown a peak value at around 15° which is predicted by the theoretical simulation. Low performance of the microhole textured solar cell is related to the enhanced surface recombination at the surface whose area is increase due to the microhole formation, and also due to the poor metal contact formation.

## Conflict of Interest

The authors declare no conflict of interest.
